# Genome-scale study reveals reduced metabolic adaptability in patients with non-alcoholic fatty liver disease

**DOI:** 10.1038/ncomms9994

**Published:** 2016-02-03

**Authors:** Tuulia Hyötyläinen, Livnat Jerby, Elina M. Petäjä, Ismo Mattila, Sirkku Jäntti, Petri Auvinen, Amalia Gastaldelli, Hannele Yki-Järvinen, Eytan Ruppin, Matej Orešič

**Affiliations:** 1 Department of Systems Medicine, Steno Diabetes Center, Niels Steensens Vej 6, Gentofte, DK-2820, Denmark; 2 VTT Technical Research Centre of Finland, Espoo, FI-02044 VTT, Finland; 3 Blavatnik School of Computer Science, Tel Aviv University, Tel Aviv 69978, Israel; 4 Department of Medicine, Division of Diabetes, University of Helsinki, Helsinki, FI-00014, Finland; 5 Minerva Foundation Institute for Medical Research, Helsinki FI-00290, Finland; 6 Faculty of Pharmacy, University of Helsinki, Helsinki FI-00014, Finland; 7 Institute of Biotechnology, DNA Sequencing and Genomics Laboratory, University of Helsinki, Helsinki FI-00014, Finland; 8 Institute of Clinical Physiology, National Research Council, Pisa 56124, Italy; 9 Center for BioInformatics and Computational Biology, Department of Computer Science, University of Maryland, College Park, Maryland 20742, USA; 10 Turku Centre for Biotechnology, University of Turku and Åbo Akademi University, Turku FI-20520, Finland

## Abstract

Non-alcoholic fatty liver disease (NAFLD) is a major risk factor leading to chronic liver disease and type 2 diabetes. Here we chart liver metabolic activity and functionality in NAFLD by integrating global transcriptomic data, from human liver biopsies, and metabolic flux data, measured across the human splanchnic vascular bed, within a genome-scale model of human metabolism. We show that an increased amount of liver fat induces mitochondrial metabolism, lipolysis, glyceroneogenesis and a switch from lactate to glycerol as substrate for gluconeogenesis, indicating an intricate balance of exacerbated opposite metabolic processes in glycemic regulation. These changes were associated with reduced metabolic adaptability on a network level in the sense that liver fat accumulation puts increasing demands on the liver to adaptively regulate metabolic responses to maintain basic liver functions. We propose that failure to meet excessive metabolic challenges coupled with reduced metabolic adaptability may lead to a vicious pathogenic cycle leading to the co-morbidities of NAFLD.

## Abstract (web summary)

Non-alcoholic fatty liver disease (NAFLD) is a risk factor for other types of liver diseases. Here, the authors integrate transcriptomic and metabolomic data from patients with NAFLD with a genome-scale metabolic model to paint a comprehensive picture of liver function in NAFLD.

## Graphical abstract

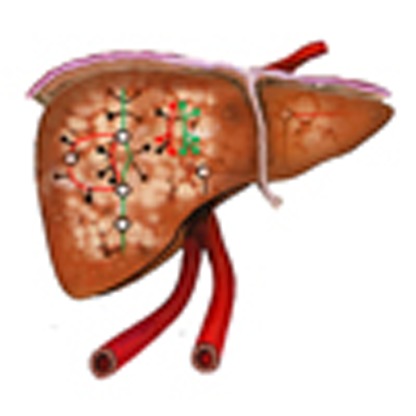

The prevalence of non-alcoholic fatty liver disease (NAFLD) in US adults doubled from 5.51 to 11.01% between 1988 and 2008 and may soon reach epidemic proportions if the current rates of obesity and diabetes continue to escalate^1^. NAFLD is a major component of the metabolic syndrome^2^,^3^,^4^, but despite its importance it is yet poorly understood. We have previously shown that fatty liver synthesizes excess triglycerides with low carbon number and double bond content^5^, indicative of increased *de novo* hepatic lipogenesis^5^,^6^. Recent research also implicates activation of liver mitochondrial pathways, *de-novo* lipogenesis and gluconeogenesis (GNG) contributing to the development of NAFLD^7^.

As it is technically demanding and highly invasive, only a few studies have investigated the human splanchnic metabolism of metabolites such as amino acids, free fatty acids (FFAs) and ketone bodies *in vivo*^8^,^9^. The main limitation of such early studies was a poor coverage of the metabolites associated with liver functions, because of the limitations in sensitivity or selectivity of the analytical methodologies available at that time. Metabolomics platforms that afford comprehensive coverage of polar metabolites and molecular lipids are now available.

Here we aim to characterize these essential metabolic pathways of the human liver on a genome scale. We harness genome-scale metabolic modelling (GSMM) to meet the challenges introduced by high-throughput and data-rich biology. GSMM has been shown to provide an appropriate context for analysing high-dimensional ‘omics’ data sets and elucidate the genotype-to-phenotype relationship^10^,^11^,^12^,^13^,^14^. Accounting for thermodynamic and stoichiometric constraints, GSMM captures metabolic phenotypes under diverse physiological and genetic conditions^13^,^14^,^15^. Algorithmic approaches have leveraged GSMM as a mechanistic scaffold for drug discovery^16^ and biomarker identification^17^. GSMM was recently applied to study NAFLD and non-alcoholic steatohepatitis (NASH)^18^. In this study, a GSMM of hepatocytes was constructed and utilized to analyse gene expression profiles of NAFLD patients. Blood concentrations of chondroitin and heparan sulphates were found suitable for diagnosing NASH and staging NAFLD. Taken together, these recent methodological developments now lay the basis for integrated experimental-computational studies that could broaden our understanding of the metabolic processes associated with the normal and pathological human liver. With the aim to analyse liver metabolism at the genome scale in response to NAFLD development, herein we obtain transcriptomics in human liver biopsies from subjects with NAFLD and controls, and—for the first time—comprehensively characterize the metabolic fluxes across the splanchnic bed in human fatty liver. The fluxes and gene expression data are integrated with a genome-scale model of human metabolism^10^,^19^ to chart liver metabolic activity in NAFLD on a genome scale aiming to identify its distinctive features (Fig. 1).

## Results

### Gene expression-based analysis of liver metabolic functionality

Gene expression was measured in the human liver of eight subjects with extreme steatosis diagnosed with NAFLD (liver fat % range 20–80%), and eight healthy subjects with low liver fat content (liver fat % range 0–10%; Supplementary Table 1). There was no significant difference between the groups in age, gender and PNPLA3 genotype at single-nucleotide polymorphism rs738409. In the high liver fat group, there were two patients who had mild fibrosis but no patients had impaired hepatic function. Subjects with high liver fat content had slightly but not significantly higher fasting glucose and HbA_1c_ concentrations than subjects with low liver fat content. They also had significantly higher liver enzyme concentrations.

The metabolic functionality of each of these patients was evaluated by inferring their capacity to carry out various liver metabolic functions, as defined by Gille *et al.*^20^. To this end, the gene expression profiles were integrated with a GSMM and transformed into metabolic functionality profiles^21^. The metabolic model used—*Homo sapiens* Recon 1—accounts for the functions of 1,496 open reading frames, 2,004 proteins, 2,766 metabolites and 3,311 metabolic and transport reactions^22^.

The metabolic functionality profile of a patient was generated in two complementary ways. First, we estimated the activity level of different metabolic functions under the constraint to maintain an optimal fit to the gene expression, by applying the integrative Metabolic Analysis Tool (iMAT)^19^,^23^. Second, we applied another GSMM approach for integrating ‘omics’ data termed metabolic phenotypic analysis (MPA)^24^. Given the gene expression profile, MPA assigns each metabolic function an adaptability score. A high adaptability score indicates that the function studied can be carried out with relatively little deviation from the given expression state; a low adaptability score denotes that in order to perform that function the cells must undergo considerable transcriptional changes or post-transcriptional regulation to enable the desired flux rerouting to be carried on (see Methods). The metabolic profiles obtained by iMAT and MPA are hence complementary representations of the metabolic state: iMAT infers the flux activity of the function at the time the gene expression was taken, whereas MPA aims to deduce the underlying *metabolic adaptability* at the network level.

**Figure 2:**
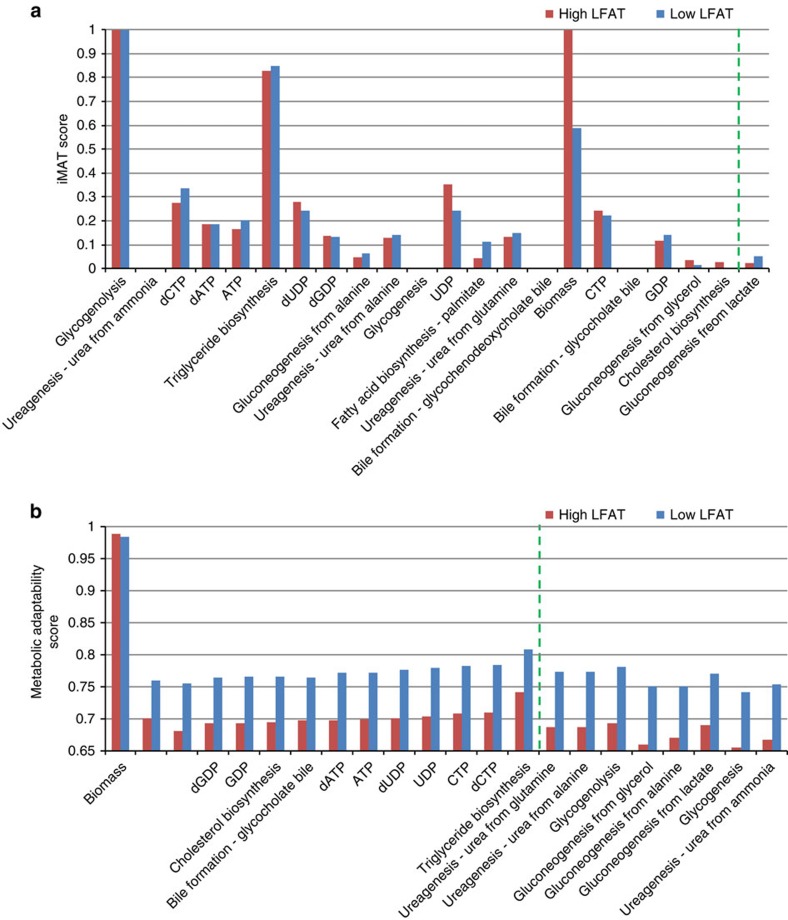
GSMM analysis reveals reduced metabolic adaptability in NAFLD. Median functionality scores (*y* axis) obtained in the high (red) and low (blue) fat groups according to: (**a**) iMAT and (**b**) metabolic adaptability analysis (MPA). The functions are ordered according to their *P*-values (Wilcoxon signed-ranksum test), from largest to smallest, such that the ones that are on the right side of the dashed line obtained *P*-values that are less than 0.1.

The GSMM functionality analysis described above showed that the NAFLD group was characterized by decreased activity of GNG from lactate as compared with the control group (Fig. 2a), that is, in NAFLD patients less lactate is converted into glucose via GNG. According to the metabolic adaptability scores, the low liver fat group consistently exhibited higher metabolic adaptability throughout the different metabolic functions versus the NAFLD group (Fig. 2b). Specifically, the NAFLD group had defective adaptability of ureagenesis and metabolic functions related to GNG (Wilcoxon signed rank sum test *P*-values<0.05).

### A genome-scale view of splanchnic fluxes in NAFLD

Next, we compared the GSMM predictions from the transcriptomics study with metabolomics data measured across human splanchnic vascular bed in an independent study. Nine subjects with NAFLD underwent hepatic venous catheterization in the fasting (basal) state and during euglycemic hyperinsulinemia^5^ (Supplementary Table 2). Global profiling of small polar metabolites^25^ and targeted profiling of bile acids^26^ were applied to study the fluxes and ratios of the metabolites in NAFLD subjects with varying levels of liver fat content (Supplementary Table 3). Although these measurements provide an opportunity to characterize metabolism in the human liver, they are limited to a small number of fluxes measured at specific uptake and secretion reactions. Nonetheless, these observed fluxes constrain considerably the flux distribution over the rest—unobserved—liver metabolic reactions.

To uncover the latter, we employed an iMAT GSMM analysis of the human metabolic model to predict the most likely flux activity state induced upon the network given the flux data measured (Supplementary Table 4). Reassuringly, the activity state inferred by the genome-scale model was consistent with the observed fluxes in a statistically significant manner (empirical *P*-value of 0.002 versus its fit to randomly generated flux data; see Methods). Furthermore, a standard leave-one-out cross-validation procedure demonstrated that the model correctly predicts the flux of a measured reaction when other flux measurements are provided (Spearman correlation *P*-value of 1.34 × 10^−11^). Thus, using this analysis of the measured—small-scale—flux data, we were able to reliably infer the metabolic activity at the level of reactions, and at the level of metabolic pathways and functions, on a genome scale.

To test whether the GSMM analysis provides a reliable platform to compare the metabolic state based on different data sources, we also computed the metabolic functionality scores, based on the flux measurements and gene expression, and utilized them as classification features. According to these features, the patients were classified as having high or low liver fat amount via Support Vector Machine (SVM)^27^. First, only the patients whose gene expression was measured were classified. The classifier that exploits the metabolic functionality scores as features obtained a significantly high mean area under the curves (AUC) of 0.857 (empirical *P*-value of 0.06). It significantly improves upon the classifier that utilizes the raw gene expression as features (mean AUC of 0.774; empirical *P*-value of 0.178). Classifying all patients according to the metabolic functionality scores also obtained a significantly high AUC of 0.79 (empirical *P*-value of 0.029). Furthermore, when training the classifier based on the metabolic functionality scores inferred from the flux measurements and testing it based on those inferred from the gene expression, the obtained AUC is 0.792 (empirical *P*-value of 0.065). These results demonstrate the potency and validity of GSMM as a unifying representation. By deducing the metabolic phenotype it enables one to bridge across different measurement types as gene expression and flux rates.

GSMM analysis of the splanchnic gradients showed that, as expected, the basal state is significantly less metabolically active compared with the euglycemic hyperinsulinamic state (Wilcoxon ranksum test *P*-value of 3.557 × 10^−4^; Supplementary Table 4). Lactate and intermediates of GNG as pyruvate and citrate were taken up by the liver (Supplementary Table 3). In addition, glutamic acid, several sugar derivatives and hydroxy-acids as ketoacids and α-hydroxybutyric acid were produced by the liver. Most of the bile acids and amino acids, including alanine and methionine, were taken up by the liver. Notably, serine, which was proposed to be decreased in the liver of NASH patients^18^, was taken up by the liver.

**Figure 3:**
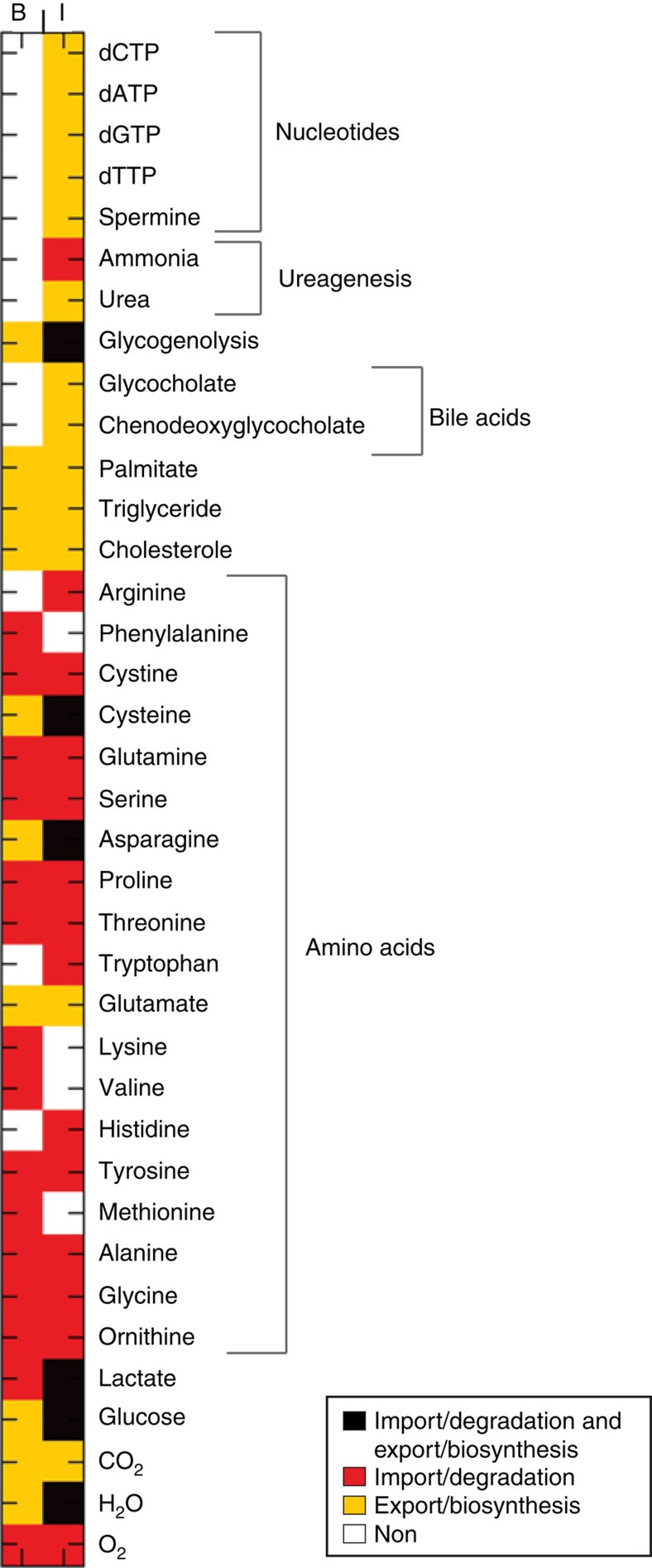
Genome-scale view of liver metabolic functionality and splanchnic fluxes in the basal state (B) and in low-dose insulin infusion (I) obtained from flux-based GSMM analysis. Metabolites that can be exported/synthesized (imported/degraded) are coloured yellow (red). If both import and export are feasible (infeasible) then the corresponding square is coloured black (white). The first and second columns correspond to the basal state and low-dose insulin infusion simulations, respectively.

As expected, the anabolic state induced by the euglycemic hyperinsulinamic clamp triggered major changes in the metabolic fluxes (Fig. 3 and Supplementary Tables 4–5). As shown in Fig. 3, fatty acids, asparagine, glutamate, cysteine, triglycerides and glucose were produced by the liver in both basal state and after insulin infusion. The insulin infusion also induced the biosynthesis of bile acids, nucleotides and glycogen, consistent with anabolic effects of insulin. Insulin administration decreased the plasma concentration of branched chain amino acids.

### Liver fat has a major effect on liver metabolic pathways

Examining the experimental flux measurements directly from measured metabolite concentration data, a large number of key metabolite fluxes were strongly associated with the amount of liver fat, particularly in the basal state (Supplementary Table 6). Also the arterial concentrations of fuel substrates were upregulated (1.4- to 1.9-fold) in those patients with very high liver fat accumulation. Specifically, lactic acid (*P*<0.001; two-sided *t*-test), pyruvic acid (*P*=0.017), FFAs (*P*=0.0012), alanine (*P*<0.001) and β-hydroxybutyrate (*P*=0.0089) were increased in patients with increased liver fat content. Splanchnic fluxes of lactic acid (Spearman correlation *R*=−0.99, *P*<0.001) and pyruvic acid (*R*=−0.997, *P*<0.001) were inversely correlated with liver fat amount in the basal state. β-Hydroxybutyrate was produced across the splanchnic bed in all patients.

Patients with higher liver fat had lower measured uptake of fatty acids and glycerol as compared with the patients with lower liver fat. On the other hand, the uptake of amino acids, particularly alanine (*P*<0.001), was higher in patients with higher liver fat. The insulin administration caused clear changes in metabolic fluxes of the patients with relatively low amount of liver fat. In contrast, patients with very high liver fat did not respond to insulin administration as there was hardly any change in metabolite fluxes associated with glycolysis, lipolysis or ketogenesis.

Flux-based GSMM analysis also confirmed that three specific processes: (i) increased activity of pathways leading to GNG and glyceroneogenesis, (ii) trichloroacetic acid (TCA) cycle and (iii) *de novo* lipogenesis were significantly increased in the patients with high amount of liver fat. In the basal state, the maximal fluxes of 39 reactions were significantly positively correlated (empirical and Spearman correlation *P*-value<0.05; with false discovery rate correction for multiple-hypotheses) with the amount of fat (Supplementary Table 7). These reactions were highly enriched with reactions of triglyceride synthesis and lipogenic processes (hyper-geometric *P*-value of 3.9 × 10^−4^).

### Type 2 diabetes leads to increased GNG from lactate/pyruvate

**Figure 4:**
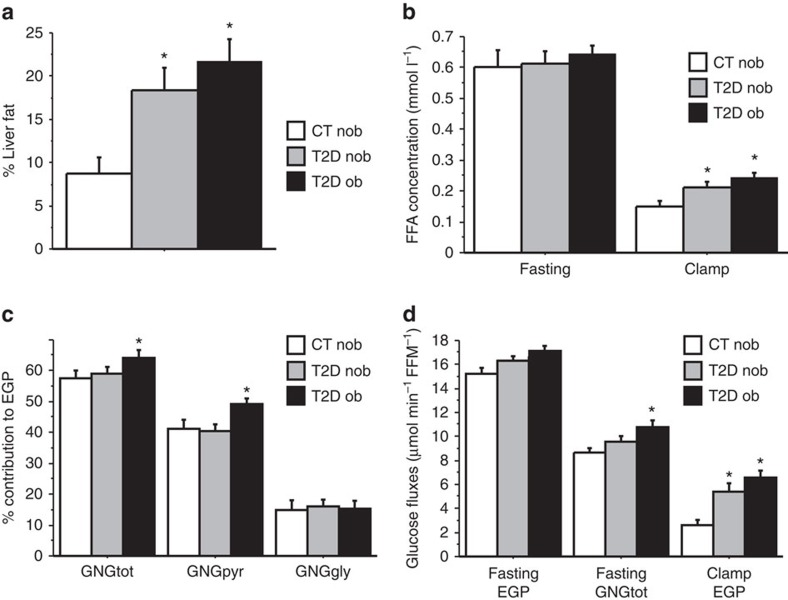
Gluconeogenesis in nonobese (nob; *N*=24) and obese (ob; *N*=19) type 2 diabetic patients (T2D) and nondiabetic (*N*=14) subjects, based on data from an earlier study^28^. (**a**) Liver fat percentage and (**b**) free fatty acid (FFA) concentrations in the three study groups. (**c**) Contributions of gluconeogenesis to endogenous glucose production. (**d**) Glucose fluxes in the three study groups.CT, non-diabetic non-obese control subjects; EGP, endogenous glucose production; FFM, fat-free mass; GNG, gluconeogenesis; GNGgly, GNG from glycerol; GNGpyr, GNG from lactate/pyruvate; GNGtot, total GNG. Data reported as mean±s.e.m., *P<0.05 (*t*-test versus CT).

Diabetes worsens the situation, as these subjects not only have more liver fat than healthy subjects but also have increased fasting glucose production and GNG, and impaired suppression of glucose production, GNG and peripheral lipolysis during insulin administration. We examined previously unreported data on relative contribution of different sources to GNG from an earlier study of diabetic and nondiabetic obese patients^28^. In diabetic patients, lactate/pyruvate and amino acids contribute to GNG more than glycerol. However, it cannot be excluded that the glyceroneogenic pathway is increased and contributes to excess triglyceride synthesis (Fig. 4).

## Discussion

**Figure 5:**
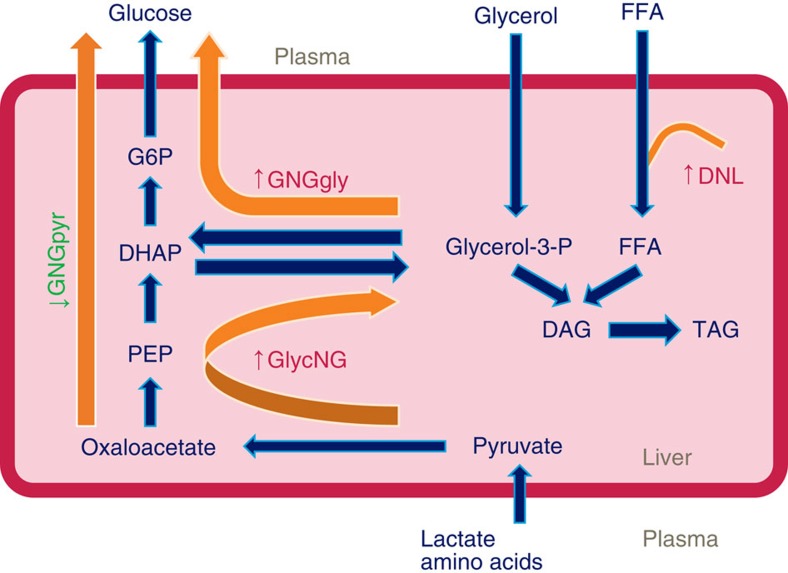
Biochemical model of regulation of glucose and triglyceride synthesis in NAFLD. Tight balance between glyceroneogenesis (GlycNG) and gluconeogenesis from glycerol (GNGgly) in NAFLD patients may help maintain the glycemic control, but at a cost of lower adaptability of the global metabolic network. This balance breaks down in type 2 diabetes, where gluconeogenesis from pyruvate (GNGpyr) is markedly increased.DAG, diacylglycerol; DHAP, dihydroxyacetone phosphate; DNL, *de novo* lipogenesis; FFA, free fatty acid; G6P, glucose-6-phosphate; PEP, phosphophenol pyruvate; TAG, triacylglycerol.

In this study, we show that the liver adaptively regulates metabolic responses to maintain its basic functions. We also show that NAFLD is associated with increased mitochondrial metabolism, lipolysis, glyceroneogenesis and a switch from lactate to glycerol as substrate for GNG. Tight balance between the increased glyceroneogenesis and GNG from glycerol may help maintain the glycemic control in nondiabetic subjects (Fig. 5), but at a cost of lower adaptability of the global metabolic network as observed by GSMM analysis. This balance clearly breaks down in type 2 diabetic subjects, where the contribution to GNG from lactate/pyruvate is markedly increased.

The increased utilization of glycerol as substrate for GNG may facilitate the development of metabolic co-morbidities of NAFLD as glycerol is the only gluconeogenic substrate that imports new carbons to the glucose pool and might therefore contribute to deterioration of glycemic control^29^,^30^. However, glycerol could also be used for triglyceride synthesis as the liver is one of the few organs that contains glycerokinase. The increased glyceroneogenesis, which may contribute to glycemic control in nondiabetic subjects by reducing the contribution of lactate to GNG (Fig. 5), may therefore come at a cost of increased triglyceride synthesis^31^ and increased GNG from glycerol.

Our key findings corroborate several of the earlier findings. Specifically, the substrate concentrations as well as fluxes of amino acids, ketone bodies and pyruvate are consistent with earlier studies describing splanchnic fluxes in obesity and various liver diseases^8^,^9^. Among them, ketogenesis, proteolysis and peripheral lipolysis were suppressed during euglycemic hyperinsulinemia. This could be seen in the decreased arterial concentration levels of precursors of these pathways, such as multiple amino acids (including branched chain amino acids) and FFAs, as well as of markers such as α- and β-hydroxybutyric acids. Notably, α-hydroxybutyric acid has been considered as an early marker for both insulin resistance and impaired glucose regulation^32^.

A tracer study of NAFLD patients and controls suggest that NAFLD is associated with increased TCA cycle, GNG and lipolysis in the liver^7^. Our study confirms that higher liver fat is associated with more active TCA cycle metabolism. However, we found in two independent approaches (flux- and gene expression-based analysis) that GNG from lactate is in fact decreased with high liver fat at the expense of increased glyceroneogenesis. Instead, patients with higher liver fat had higher rates of lipolysis and GNG from glycerol. These differences were not due to obesity itself as all patients were morbidly obese. Understandably, we were not able to obtain liver biopsies from lean healthy subjects as such would be unethical. The patients who participated in the catheterization study were all patients who had been referred to the hepatologist for a liver biopsy because of suspected NASH. These patients were significantly less obese than the patients in the transcriptomics study. This is a limitation of the present study.

Together, our findings indicate that in NAFLD glycerol is being utilized as a preferential substrate for GNG instead of lactic acid. In support of this hypothesis, 3-glycerolphosphate, an intermediate of lipolysis, as well as multiple FFAs were positively correlated with liver fat in the basal state. Furthermore, GSMM analysis utilizing the metabolite flux data as constraints showed that reactions of glycerol kinase and glycerol-3-phosphate acyltransferase, two of the key enzymes for glyceroneogenesis and triglyceride synthesis, are strongly associated with liver fat deposition. In support of our observation of the increased activity of glycerol kinase, its protein abundance was found to be significantly increased in animal models with fatty liver^33^. Moreover, a deficiency of mitochondrial glycerol-3-phosphate acyltransferase results in reduced body weight and liver triacylglycerol content and altered glycerolipid fatty acid composition^34^, whereas overexpression of this enzyme results in hepatic steatosis^35^.

Large differences were observed in the metabolic adaptability scores when comparing NAFLD patients and healthy controls, implying that NAFLD is characterized by reduced metabolic adaptability at a network level. In other words, maintaining its normal functioning requires a burden of post-transcriptional regulation to increase as NAFLD progresses. This is consistent with our finding that NAFLD is associated with increased opposite metabolic processes such as glyceroneogenesis and GNG from glycerol, which may together contribute to the glycemic control. Such a fine balance of metabolic processes is broken down in type 2 diabetes, where the GNG from lactate is markedly increased. According to our model, high liver fat markedly hampers the ability of the liver to adaptively regulate metabolism to meet excessive demands on basic liver functions. Therefore, individuals with NAFLD may be more vulnerable to various metabolic stressors on the liver, such as hypercaloric diet or other lifestyle factors contributing to positive energy balance. Taken together, our data are consistent with NAFLD imposing a burden on hepatic intermediary metabolism and suggest that specific dietary regimes or therapies releasing pressure on the metabolic switch observed in our study may ameliorate disease progression in patients with NAFLD.

In summary, our study clearly shows that the increasing fat load has a systemic effect on liver metabolic networks such as reduced metabolic adaptability as well as induces mitochondrial metabolism, glyceroneogenesis and a switch from lactate to glycerol as substrate for GNG. The increasing metabolic stress and loss of metabolic adaptability may together contribute to the vicious pathogenic cycle leading to the co-morbidities of NAFLD including development of type 2 diabetes.

## Methods

### Subjects in transcriptomics study

Clinical characteristics of the study groups are shown in Supplementary Table 1. The subjects represented low and high extremes of liver fat content of a group of previously described subjects undergoing laparoscopic gastric bypass surgery or who were referred to the gastroenterologist because of elevated liver function tests, and from patients whose remaining liver samples sufficient amounts of high-quality RNA could be prepared^36^. In brief, these patients fulfilled the following inclusion and exclusion criteria: (i) age 18–60 years; (ii) alcohol consumption less than 2 drinks/day and (iii) no evidence of hepatitis B or C, thyroid dysfunction, autoimmune hepatitis (smooth muscle and antinuclear antibodies), primary biliary cirrhosis (antimitochondrial antibodies), primary sclerosing cholangitis, α_1_-antitrypsin deficiency, use of hepatotoxic medications or herbal products, or use of medications known to be associated with steatohepatitis. Liver histology was analysed by an experienced liver pathologist in a blinded manner as proposed by Brunt *et al.*^37^. Fat content of the liver biopsy specimens was evaluated as % hepatocytes with macroscopic steatosis.

The nature and potential risks of the study were explained to all subjects before obtaining their written, informed consent. The study was carried out in accordance with the principles of the Declaration of Helsinki. The protocol was approved by the ethics committee of the Helsinki University Central Hospital.

### Subjects in metabolomics study

Characteristics of the study subjects are shown in Supplementary Table 2. All subjects were recruited among subjects referred to a gastroenterologist because of elevated liver function tests based on the following inclusion criteria: (i) age 18–75 years; (ii) no known acute or chronic disease except for obesity based on history, physical examination and standard laboratory tests (blood counts, serum creatinine, thyroid-stimulating hormone and electrolyte concentrations) and ECG; (iii) alcohol abuse. The following causes of liver diseases were excluded: chronic hepatitis B or C, thyroid dysfunction, autoimmune hepatitis (smooth muscle and anti-nuclear antibodies), primary biliary cirrhosis (anti-mitochondrial antibodies), primary sclerosing cholangitis, use of hepatotoxic medications or herbal products or use of medications known to be associated with steatohepatitis. Three subjects were receiving medications for hypertension. The nature and potential risks of the study were explained to all subjects before obtaining their written informed consent. The study protocol was approved by the ethics committee of the Karolinska Hospital (Stockholm, Sweden). For ethical and technical reasons, it was not possible to perform a similar study in healthy volunteers.

### Design of the metabolomics study

The subjects were studied after an overnight fast. Three intravenous cannulas were inserted as described earlier^5^. Shortly, the first was inserted in an antecubital vein for infusions of saline (first 90 min), glucose, insulin and potassium-[2,2-^2^H_2_]palmitate, the second was inserted in a contralateral arm and the third catheter was inserted percutaneously under local anaesthesia into a femoral vein^38^,^39^. Hepatic blood flow was measured using a constant intravenous infusion of indocyanine green^40^. Plasma glucose was maintained at 5 mmol l^−1^ (90 mg dl^−1^) until 240 min using a variable rate infusion of 20% glucose. Potassium-[2,2-^2^H_2_]palmitate bound to human albumin was infused at a rate of 0.05 μmol kg^−1^min^−1^ starting at 30 min to trace FFA and triglyceride metabolism *in vivo*. Hepatic blood flow was measured at 90, 100, 110, 120, 200, 220, 240 min. During the saline and insulin infusion, arterialized and hepatic vein plasma samples were taken at 10–60 min intervals.

The fat content of the liver biopsy specimens (% of hepatocytes with macrovesicular steatosis) was determined by an experienced liver pathologist in a blinded manner. The % of macrovesicular steatosis was used as the liver fat %. The clinical parameters shown in Supplementary Table 2 were reported previously^5^.

### *In vivo* study of gluconeogenic pathways

In another study conducted in 14 nondiabetic and 24 lean and 19 obese type 2 diabetic subjects^28^, liver fat content was measured by magnetic resonance spectroscopy. We measured endogenous (hepatic) glucose production (by ^3^H-glucose infusion) and the contribution of GNG (by ^2^H_2_O ingestion) in the basal state and during 240 pmol m^−2^ min^−1^ euglycemic-hyperinsulinemic clamp. Percent contribution of GNG to endogenous (hepatic) glucose production (GNGtot) was quantified by the ratio of enrichments of deuterium bound at Carbon 5 and at Carbon 2 of glucose molecule^41^; GNGpyr, that is, from lactate/pyruvate, as the ratio of enrichments of deuterium bound at Carbon 6 and at Carbon 2 (ref. 42); and GNGgly, that is, from glycerol, as the difference between GNGtot and GNGpyr.

### Transcriptomic analysis of liver biopsies

The transcriptomic analysis was performed as in the earlier study at the DNA Sequencing and Genomics laboratory at the University of Helsinki (Helsinki, Finland)^43^. In short, microarray analysis was done using Agilent Whole Human Genome Microarray Kit, 4x44K chips (Agilent catalogue # G4112F). Image analysis to compute the intensity values from the TIFF files was done using GenePix Pro 6.0 software. The background was estimated using local features, and bad spots were flagged manually.

The individual channel intensities were read into R/Bioconductor^44^ and custom spot quality weights were assigned. The intensity data are then processed with quantile normalization method^45^. The preprocessing was performed using limma package^46^ of R/Bioconductor.

### Metabolomic analysis

Metabolomic analysis was performed at VTT Technical Research Centre of Finland (Espoo, Finland).

*Global analysis of polar metabolites*. Metabolites were measured by the established analytical platform based on two-dimensional gas chromatography coupled to time-of-flight mass spectrometry (GC × GC-TOFMS)^25^. Each serum sample (20 μl) was spiked with internal standard (20 μl labelled palmitic acid, *c*=258 mg l^−1^) and the mixture was then extracted with 400 μl of methanol. After centrifugation, the supernatant was evaporated to dryness and the original metabolites were then converted into their methoxime and trimethylsilyl derivative(s) by two-step derivatization. First, 25 μl methoxamine reagent was added to the residue and the mixture was incubated for 60 min at 45 °C. Next, 25 μl *N*-methyl-*N*-(trimethylsilyl) trifluoroacetamide was added and the mixture was incubated for 60 min at 45 °C. Finally, retention index (RI) standard mixture (n-alkanes) in hexane was added to the mixture.

For the analysis, a Leco Pegasus 4D GC × GC-TOFMS instrument (Leco Corp.) equipped with a cryogenic modulator was used. The GC part of the instrument was an Agilent 6890 gas chromatograph (Agilent Technologies), equipped with split/splitless injector. The first-dimension chromatographic column was a 10-m RTX-5 capillary column with an internal diameter of 0.18 mm and a stationary-phase film thickness of 0.20 μm, and the second-dimension chromatographic column was a 1.5-m BPX-50 capillary column with an internal diameter of 100 μm and a film thickness of 0.1 μm. A diphenyltetramethyldisilazane deactivated retention gap (3 m × 0.53 mm i.d.) was used in the front of the first column. High-purity helium was used as the carrier gas at a constant pressure mode (39.6 psig). A 5-s separation time was used in the second dimension. The mass spectra were recorded by electron ionization at 70 eV in the *m*/*z* range of 45–700 a.m.u. with 100 spectra s^−1^. Split injection (1 μl, split ratio 1:20) at 260 °C was used. The temperature programme was as follows: the first-dimension column oven ramp began at 50 °C with a 1-min hold after which the temperature was programmed to 295 °C at a rate of 10 °C min^−1^ and then held at this temperature for 3 min. The second-dimension column temperature was maintained 20 °C higher than the corresponding first-dimension column. The programming rate and hold times were the same for the two columns.

The original GC × GC-TOFMS data included retention times, RIs, spectral information, possible identification, spectral similarity value (*S*=0–1,000) and peak response data. The linear RIs were calculated based on the retention times of the compounds and the retention times of the RI standards (n-alkanes). The data were transferred into an in-house developed programme Guineu^25^. The alignment of the data was done based on the two retention times and spectra. After alignment of the GC × GC-TOFMS data, two filtration criteria were used for positive identification: (i) spectral match <850 and (ii) the RIexp-RIlit<25 or RIexp-RIstd_exp<25, in which RIexp is the experimental RI for a compound and RIlit is the literature value for the identified compound and RIstd_exp is the experimental RI value for a standard compound. Compounds not fulfilling the criteria were renamed to unknowns and were subjected to further identification.

The identification was based on the spectral search from NIST library or the in-house collected library and the RIs. The compounds for which no spectral match was found by the two libraries, GOLM database (http://gmd.mpimp-golm.mpg.de) was utilized. The database was also used for functional group prediction of the metabolites.

Calibration curves (six points, *c*=15–2,000 ng, cholesterol *c*=170–6,000 ng) were constructed for the following compounds: pyruvic acid, alanine, 3-hydroxybutyric acid, valine, leucine, isoleucine, proline, glycine, succinic acid, fumaric acid, serine, threonine, malic acid, methionine, aspartic acid, alpha-ketoglutaric acid, phenylalanine, glutamic acid, ornithine, citric acid, tyrosine, palmitic acid, linoleic acid, oleic acid, stearic acid, arachidonic acid and cholesterol.

Quality control of the method showed that the day-to-day repeatability of control serum samples, and the relative standard deviation (RSD) values for quantified compounds (amino acids and carboxylic acids) was on average below 10%, ranging from 3 to 17%. The repeatability was also studied for the same control serum samples using peak areas of the semiquantified compounds, and including all major peaks in the investigation (220 peaks). The average RSD for peak areas, utilizing total ion current trace, was below 24%. The internal standards added to all serum samples in the study had an average RSD under 10%.

*Analysis of bile acids*. Glycolithocholic acid (GLCA) and glycoursodeoxycholic acid (GUDCA) were obtained from Calbiochem, glycodeoxycholic acid (GDCA) and ursodeoxycholic acid (UDGA) from Fluka, cholic acid (CA), deoxycholic acid (DCA), glycocholic acid (GCA), glycochenodeoxycholic acid (GCDCA), lithocholic acid (LCA), taurocholic acid (TCA), taurochenodeoxycholic acid (TCDCA), taurodeoxycholic acid (TDCA), taurolithocholic acid (TLCA) and tauroursodeoxycholic acid (TUDCA) were from Sigma-Aldrich and α-muricholic acid (αMCA), βMCA, ωMCA, TαMCA, TβMCA and taurohyodeoxycholic acid (THDCA) from Steraloids. Internal standards, CA-d4, LCA-d4, UDCA-d4, CDCA-d4, DCA-d4, GCA-d4, GLCA-d4, GUDCA-d4, GCDCA-d4 and GDCA-d4, were obtained from Qmx laboratories except TCA-d4 was synthesized in our laboratory.

Internal standards (20 μl of standard mixture; 2 p.p.m. for TCA-d4, 0.2 p.p.m. for glycine conjugates, 2 p.p.m. for intact bile acids, respectively) and 20 μl of 1 mM norvaline was added to 20 μl sample and mixture was vortexed for 30 s. 60 μl of acetonitrile was added to precipitate proteins. After vortexing for 60 s, samples were centrifuged at 13,200 r.p.m. for 5 min. 90 μl of sample was evaporated with nitrogen and reconstituted to 20 μl of methanol, and diluted with 20 μl of water.

Analysis was performed on an Acquity Ultra-Performance Liquid Chromatography system, Waters, and Waters Xevo TQ MS. Chromatography was performed using an Acquity HSS T3 (2.1 × 150 mm^2^, 1.7 μm) column, Waters, kept at 30 °C. Injection volume was 2.0 μl. Separation was performed using (i) gradient elution with 10 mM formic acid in water (v/v) and (ii) with 10 mM formic acid in acetonitrile at a flow rate of 0.4 ml min^−1^ with a gradient elution mode. Mass spectrometry was performed in negative ion electro spray mode. Cone voltage was 70 V and collision energy 2 eV for intact bile acids, 58 V and 46 eV for glycine conjugates and 90 V and 63 eV for taurine conjugates, respectively. The capillary voltage was 2.4 kV, desolvation temperature was 650 °C and source temperature was 150 °C. The cone gas flow was 30 l h^−1^, desolvation gas was 1,000 l h^−1^ and collision gas was 0.15 ml min^−1^. Analytes were detected using selected reaction monitor using dwell times of 100–250 ms.

### Human model modification

The modification of the human GSMM^10^ includes the addition of a biomass reaction and two reactions that enable the formation and degradation of glycogen. These reactions are: (i) the demand of glycogen and (ii) the demand of apo-glycogenin protein, the primer for glycogen synthesis. The modified model in a MATLAB format is accessible through a supplementary website http://www.cs.tau.ac.il/~livnatje/NAFLD_model.mat.

### Expression-based GSMM analysis and metabolic functionality assessment

Gene expression profiles were analysed via GSMM to generate metabolic profiles for each patient. These profiles aim to capture the metabolic phenotype by integrating the gene expression profiles with a GSMM^21^. The metabolic profiles include an activity and an adaptability score for 22 key liver metabolic functions as defined by Gille *et al.*^20^. A metabolic function is defined by a specific reaction—usually a reaction that generates the end-product of the metabolic function—and a set of constraints. For example, for the process of GNG from lactate, the constraints are defined such that lactate is the only carbon source that can be used to produce glucose, and the end-reaction is the one that produces glucose.

Given the gene expression profile of sample *j* the activity score of a metabolic function *i* is computed as follows. Let *r*_*i*_ and *C*_*i*_ be the reaction and set of constrains that define metabolic function *i*. Let *A*_*i*_ be the maximal flux through *r*_*i*_ when the model is constrained according to *C*_*i*_. Let *A*_*ij*_ be the maximal flux through *r*_*i*_ when the model is constrained according to *C*_*i*_ and according to the gene expression of sample *j*. The gene expression constraints are imposed by applying iMAT^19^,^47^. The activity score of a metabolic function *i* in sample *j* is then the ration *A*_*ij*_*/A*_*i*_. The activity sore, hence, denotes the ability of the model to activate the metabolic function while maintaining the optimal consistency to the gene expression.

The adaptability score of a metabolic function *i* in sample *j* is computed by employing MPA^48^. Let *Fa*_*ji*_ be the maximal fit to the gene expression under the *C*_*i*_ constraints when enforcing *r*_*i*_ to be active. *r*_*j*_ is activated by setting its lower bound to be 0.5 of its maximal flux rate in the original—unconstrained—model. Let *F*_*ji*_ be the maximal fit to the expression under the *C*_*i*_ constraints. The ration *Fa*_*ji*_/*F*_*ji*_ is the adaptability score of the metabolic function *i* in a sample *j*. High adaptive functionality values indicate that the function studied can be carried out with relatively little deviation from the given expression state and vice versa.

The MATLAB implementation of the metabolic functionality assessment is accessible through a supplementary website http://www.cs.tau.ac.il/~livnatje/MPA.zip.

### Flux-based GSMM analysis

The flux-based analysis was conducted in a two-staged manner by utilizing a modified version of the human GSMM^10^. Primarily, given a set of fluxes, a feasible flux distribution that optimally fits the flux measurements and satisfies stoichiometric and thermodynamic constraints is found. This is done by solving a quadratic programming (QP) problem in which the Euclidian distance between the measurements and the resulting predicted flux distribution is minimized. This minimal distance is termed the consistency score, as it denotes the consistency between the model and the measurements. Most frequently, because of inaccuracies in the model as well as noise in the data, there is not a feasible flux solution that precisely fits the measured fluxes. Therefore, the consistency score is greater than zero. The flux distribution that is found via QP is utilized to constrain the model according to the flux measurements. Next, Flux Variability Analysis^49^ is applied to compute the upper and lower bounds of all the reactions in the model, whereas fixing the flux through the measured reactions to the values found using QP in the first step.

Based on the flux intervals computed above the reactions are divided into active and inactive reactions. A reaction is considered active if it can carry a flux with an absolute value greater than 0.001. Metabolic pathway activity is deduced by performing hyper-geometric enrichment tested based on the activity state of the reactions, identifying which pathways are enriched with active or inactive reactions. To identify metabolic reactions that are significantly correlated with fat-level pre- and post-insulin Spearman correlation was computed. Subsequently, an empirical *P*-value was computed by randomly shuffling the labels of the fat levels and computing the Spearman *P*-value for each reaction. The correction for multiple hypothesis testing was performed via false discovery rate control, with a rate of 0.1.

### Model consistency validation

To validate the fit between the metabolic model^10^ and the flux measurements, we utilized two data sets of 21 mean flux measured pre- and post-insulin administration. Their consistency scores were computed, and the statistical significance of these scores was evaluated as follows: 1,000 random sets of measurements, within the scale of the original flux measurements, were generated. For each one of these sets the consistency score was computed, to obtain the empirical *P*-value of the consistency scores over the real data sets.

### Metabolic functionality-based classification

The metabolic functionality scores, generated based on the gene expression measurements, were utilized to classify the patients as having a high or low liver fat amount. The classification was performed via the SVM library (LIBSVM)^50^. The samples were randomly divided into test and train sets 100 times. Each time two-third of the samples are used for training and the remaining one-third for testing. The classification performances were measured in a standard manner based on the AUCs of the receiver operating curves obtained. The statistical significance of the AUCs obtained was computed via permutation tests, performing the classification task 1,000 times with shuffled labels.

## Additional information

**Accession codes:** Gene expression data have been deposited in ArrayExpress under accession code E-MTAB-4231. Metabolomics data have been deposited in MetaboLights under accession code MTBLS298.

**How to cite this article**: Hyötyläinen, T. *et al.* Genome-scale study reveals reduced metabolic adaptability in patients with non-alcoholic fatty liver disease. *Nat. Commun.* 7:8994 doi: 10.1038/ncomms9994 (2016).

## Supplementary Material

Supplementary Information Supplementary Tables 1-7.

## Figures and Tables

**Figure 1:**
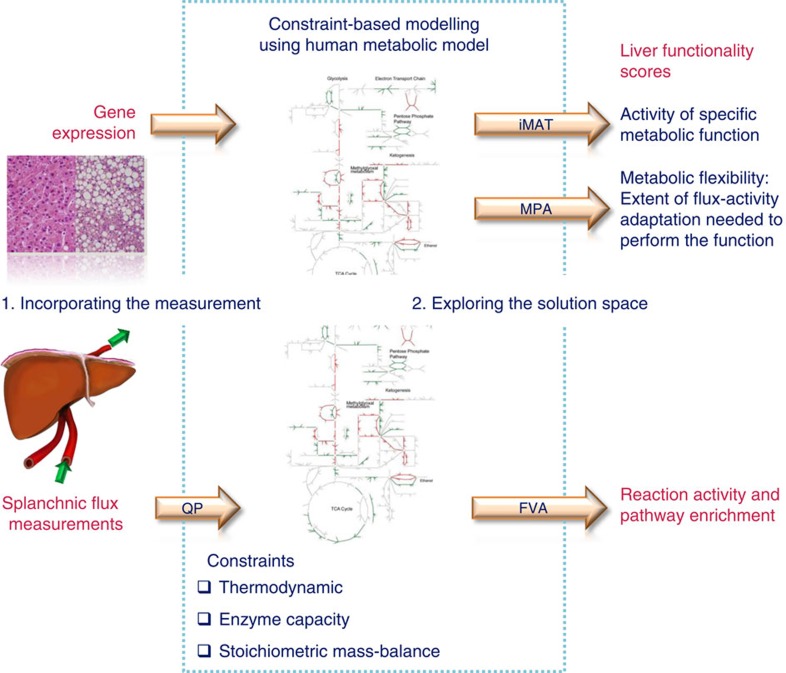
Design of the study. Genome-scale human metabolic model is integrated with the liver gene expression profile (human liver biopsies from subjects with NAFLD and controls) and the splanchnic flux (metabolomics of human serum from hepatic vein and artery from subjects with NAFLD) data by using GSMM. The GSMM analysis provides liver functionality scores, reaction activity and pathway enrichment in the context of NAFLD.FVA, flux variability analysis; iMAT, Integrative Metabolic Analysis Tool; MPA, metabolic phenotypic analysis; QP, quadratic programming.
