# Supplementary material for: Genome-scale study reveals reduced metabolic adaptability in patients with non-alcoholic fatty liver disease
Source: Nat Commun. 2016 Feb 3;7:8994. doi: 10.1038/ncomms9994 (PMC4742839; doi:10.1038/ncomms9994)
Supplement: Supplementary Information — Supplementary Tables 1-7. [file ncomms9994-s1.pdf]

Supplementary Table 1. Physical and biochemical characteristics of patients in the transcriptomics study.

	Low Liver Fat	High Liver Fat	*P*-value
n (women)	8 (4)	8 (5)	ns
Age (years)	46 ± 3	41 ± 4	ns
PNPLA3 genotype* (CC/CG/GG)	5/1/0	3/3/1	ns
**Body composition**			
Weight (kg)	153 ± 10	146 ± 7	ns
BMI (kg/m^2)	51.8 ± 2.4	49.0 ± 1.1	ns
**Laboratory parameters**			
fP-Glucose (mmol/l)	5.4 ± 0.4	6.9 ± 0.8	ns
fS-Insulin (mU/l)	13 ± 3	18 ± 4	ns
HbA$_{1c}$ (%)	5.4 ± 1.0	5.9 ± 0.3	ns
S-ALT (U/l)	25 ± 4	53 ± 7	< 0.01
S-AST (U/l)	25 ± 2	39 ± 4	< 0.01
S-γGT (U/l)	28 ± 6	40 ± 11	ns
P-Albumin (g/l)	37 ± 2	39 ±3	ns
fS-Triglycerides (mmol/l)	1.8 ± 0.3	2.1 ± 0.3	ns
fS-HDL cholesterol (mmol/l)	1.3 ± 0.1	1.0 ± 0.1	< 0.05
fS-LDL cholesterol (mmol/l)	2.3 ± 0.3	2.4± 0.3	ns
**Liver histology**			
Macrovesicular fat (%)	2 ± 1 (range 0-10)	41 ± 7 (range 20-80)	< 0.001
NASH (%)	0%	14%	ns
Stage (n, 0/1/2/3/4)	7/0/1/0/0	6/2/0/0/0	ns

Data shown as mean ± s.e.m.

*at rs738409. Data are missing from two subjects in the low and one subject in the high liver fat group.

Abbreviations: ALT, alanine aminotransferase; AST, aspartate aminotransferase; fP, fasting plasma; fS, fasting serum; NASH, non-alcoholic steatohepatitis, P, plasma; S, serum.

Supplementary Table 2. Clinical characteristics of the study subjects from the metabolomics study.

	**All subjects**
n (women)	9 (1)
Age (years)	54 ± 5
**Body composition**	
Weight (kg)	93 ± 4
BMI (kg/m^2)	29.6 ± 1.1
**Laboratory parameters**	
fP-Glucose (mmol/l)	7.1 ± 1.0
fS-Insulin (mU/l)	20 ± 4
HbA_{1c} (%)	5.7 ± 0.7
S-ALT (U/l)	92 ± 14
S-AST (U/l)	56 ± 8
S- γGT (U/l)	140 ± 40
P-Albumin (g/l)	40 ± 1
fS-Triglycerides (mmol/l)	2.7 ± 1.0
fS-HDL cholesterol (mmol/l)	1.2 ± 0.1
fS-LDL cholesterol (mmol/l)	3.4 ± 0.3
**Liver histology**	
Macrovesicular fat (%)	52 ± 26 (range 5-80)
NASH (%)	67%
Stage (n, 0/1/2/3/4)	1/3/2/3/0

Data are mean ± s.e.m.

Abbreviations: ALT, alanine aminotransferase; AST, aspartate aminotransferase; fP, fasting plasma; fS, fasting serum; NASH, non-alcoholic steatohepatitis; P, plasma; S, serum.

Supplementary Table 3. Significantly changing metabolite ratios (hepatic vein *vs*. artery; two-sided *t*-test) in the basal state or in low-dose insulin infusion based on the measurements, as obtained by metabolomics. Data for all measured bile acids is shown.

**Basal state**		**Low-dose insulin infusion**		
***P*-value**	**fold**	***P*-value**	**fold**	**Metabolite name**
**0.021**	-1.12	0.233	-1.05	Glycine
**0.040**	1.19	0.325	1.30	2-Ketovaline
**0.052**	-5.53	**0.004**	-1.98	Glutamine
0.054	-1.90	**0.017**	-1.21	Methionine
0.055	-2.03	**0.001**	-3.25	Alanine
0.059	1.93	**<0.001**	2.34	Glutamic acid
0.065	-1.74	**0.019**	-1.43	Threonine
0.065	-1.21	**0.014**	-1.29	L-Proline, hydroxy
0.080	-1.38	**0.003**	-1.21	Tyrosine
0.303	1.05	**0.006**	1.03	Pyroglutamic acid
0.446	-2.16	**0.012**	-1.55	l-Threonine
**0.728**	-1.08	**0.005**	1.41	Isoleucine
**0.034**	-1.03	0.957	1.05	Butane, 2,3-hydroxy
0.465	1.10	**0.032**	1.53	3-Oxobutanoic acid
**0.056**	1.03	**0.038**	1.19	α-Hydroxybutyric acid
**0.059**	1.35	**0.041**	1.23	β-Hydroxybutyric acid
0.200	2.34	**0.028**	-2.08	Butanoic acid, 2-methyl-3-hydroxy
0.317	1.11	**0.035**	1.14	(R*,S*)-3,4-Dihydroxybutanoic acid
0.933	1.09	**0.001**	1.46	Alpha-ketoglutaric acid
0.301	-1.01	**0.005**	1.14	b-Sitosterol
0.554	1.04	**0.049**	1.10	2-O-Glycerol-à-d-galactopyranoside
0.836	1.09	**0.015**	2.09	1H-Indole-3-acetamide
**0.005**	-1.05	**0.001**	-1.51	Cyclopentanecarboxylic acid, 1-amino-
**<0.001**	-1.08	0.193	-1.04	Linoleic acid
**0.014**	-1.73	**0.014**	-1.72	Lauric acid

**0.023**	-1.64	0.152	-1.20	2-Monopalmitin
**0.025**	-2.39	**0.012**	-1.91	9-Tetradecenoic acid
0.422	-1.18	**0.017**	-1.54	Octanoic acid
0.436	-1.11	**0.002**	1.12	Propanoic acid, 2,3-hydroxy
0.620	-1.98	**0.019**	-1.89	9-Octadecenoic acid, 2-ethyl hydroxyl ester
**0.048**	-1.69	**<0.001**	-1.51	Glycerol
**0.030**	-2.34	**0.027**	-1.45	1-Monooleoylglycerol
**0.032**	1.04	**0.011**	1.08	Myo-Inositol
**0.033**	-1.03	0.410	-1.02	Arabinofuranose
0.115	-2.00	**0.028**	-3.31	Arabitol
**0.030**	-1.06	**0.082**	-1.15	4-hydroxy-4-phenylbut-1-ene
**0.033**	-1.29	0.332	-1.15	GDCA
0.306	-1.10	0.337	-1.16	GCDCA
0.212	1.67	0.534	-1.60	GCA
**0.048**	-1.71	0.948	-1.50	GUDGA
0.723	-1.16	0.325	-1.14	CDCA
0.107	-1.21	0.553	-1.46	TCA
0.259	-1.15	0.482	-1.30	TDCA
1.000	-1.14	0.308	1.00	GLCA
0.067	-2.07	0.359	-1.14	UDCA
0.142	-1.52	0.179	-1.20	DCA

Supplementary Table 4. Enriched pathways in the basal state and in low-dose insulin infusion state. 1 and -1 denote metabolic pathways that are enriched with active or inactive reactions, respectively (hyper-geometric *P*-value <0.01). 0 denotes pathways that are not enriched neither with active nor with inactive reactions.

Pathway	Basal state	Low-dose insulin infusion	Pathway	Basal state	Low-dose insulin infusion
Exchange	1	-1	Limonene and pinene degradation	0	-1
Alanine and Aspartate Metabolism	0	1	Lysine Metabolism	1	-1
Aminosugar Metabolism	-1	1	Methionine Metabolism	1	0
Arginine and Proline Metabolism	0	1	Miscellaneous	1	1
Bile Acid Biosynthesis	-1	-1	N-Glycan Biosynthesis	-1	1
Biotin Metabolism	-1	1	N-Glycan Degradation	-1	-1
beta-Alanine metabolism	0	1	Nucleotides	1	1
C5-Branched dibasic acid metabolism	1	0	Oxidative Phosphorylation	1	1
CYP Metabolism	-1	-1	Pentose Phosphate Pathway	0	1
Carnitine shuttle	-1	1	Phenylalanine metabolism	0	-1
Cholesterol Metabolism	-1	1	Purine Catabolism	-1	1
Citric Acid Cycle	1	1	Pyrimidine Biosynthesis	-1	1
CoA Biosynthesis	0	1	Pyrimidine Catabolism	-1	0
Cysteine Metabolism	0	-1	Pyruvate Metabolism	1	1
Eicosanoid Metabolism	-1	-1	R Group Synthesis	0	1
Exchange	-1	-1	Sphingolipid Metabolism	-1	1
Fatty acid activation	0	-1	Starch and Sucrose Metabolism	-1	0
Fatty acid elongation	0	1	Taurine and hypotaurine metabolism	0	-1
Fatty acid oxidation	-1	1	Tetrahydrobiopterin	1	0
Fatty acid oxidation, peroxisome	-1	0	Thiamine Metabolism	0	1
Folate Metabolism	-1	1	Transport, Endoplasmic Reticular	0	-1

Fructose and Mannose Metabolism	-1	1	Transport, Extracellular	1	0
Galactose metabolism	0	-1	Transport, Lysosomal	-1	1
Glutamate metabolism	0	1	Transport, Mitochondrial	1	1
Glutathione Metabolism	0	1	Transport, Nuclear	1	1
Glycerophospholipid Metabolism	-1	1	Transport, Peroxisomal	-1	0
Glycine, Serine, and Threonine Metabolism	0	1	Triacylglycerol Synthesis	-1	0
Glycolysis/Gluconeogenesis	1	1	Tryptophan metabolism	0	1
Glyoxylate and Dicarboxylate Metabolism	-1	0	Tyrosine metabolism	0	-1
Heme Biosynthesis	-1	-1	Urea cycle/amino group metabolism	0	1
Hyaluronan Metabolism	0	1	Vitamin A Metabolism	1	0
IMP Biosynthesis	-1	1	Vitamin B6 Metabolism	-1	0
Inositol Phosphate Metabolism	-1	1	Vitamin D	0	-1

Supplementary Table 5. Significant metabolite concentration changes in the low-dose insulin infusion *vs*. the basal state (two-sided *t*-test) based on the measurements, as obtained by metabolomics. Data for all measured bile acids is shown.

**Artery**		**Vein**		
***P*-value**	**fold**	***P*-value**	**fold**	**Name**
**0.001**	-1.72	0.003	-1.72	4-Methyl-2-oxovaleric acid
**0.001**	0.00	0.139	0.00	Methyl cis-2-trimethylsilyl-cyclopropane-1-carboxylate
**0.006**	-1.23	0.289	-1.11	Stearic acid
**0.006**	-1.31	0.549	1.12	9-Tetradecenoic acid
**0.006**	-1.39	**0.003**	-1.16	Methionine
**0.007**	3.12	**0.020**	1.38	Butanoic acid, 2-methyl-3-hydroxy
**0.008**	2.86	0.297	-1.62	Acetic acid, hydroxyl
**0.008**	-1.14	0.061	-1.04	Isoleucine
**0.009**	-1.31	0.361	-1.07	4-hydroxy-4-phenylbut-1-ene
**0.010**	-2.62	0.684	-1.34	1H-Indole, 2,6-dimethyl-
**0.011**	-1.14	0.346	-1.11	Valine
**0.019**	-1.18	0.702	-1.04	Linoleic acid
**0.021**	1.15	0.304	1.05	1-Dimethyl(isopropyl)silyloxypropane
**0.021**	-1.21	0.522	-1.13	Hydroxyproline
**0.022**	-1.28	0.737	1.08	Butanoic acid, 2-methyl-3-hydroxy
**0.022**	-1.11	0.281	-1.11	Palmitic acid
**0.022**	-1.17	0.176	-1.19	Oleic acid
**0.023**	-1.06	0.244	-1.13	Arachidonic acid
**0.023**	-1.13	0.074	-1.07	Leucine
**0.024**	-2.05	0.823	1.62	1-Methyl-1H-1,2,4-triazole
**0.025**	-2.17	0.069	-2.48	β-Hydroxybutyric acid
**0.027**	-1.32	**0.011**	-1.16	α-Hydroxybutyric acid
**0.028**	-1.18	0.211	1.05	1,3-Dioxolane
**0.028**	-1.08	0.202	-1.07	Xylitol
**0.029**	1.43	0.855	1.02	Azelaic acid
**0.031**	-1.12	0.377	-1.19	Proline, 4-hydroxy-, trans-

**0.031**	-1.02	0.768	-1.00	2,2-Dimethyl-1-(2,4,6-trimethylphenyl)propan-1-one
**0.031**	1.27	0.285	1.13	Succinic acid
**0.035**	-3.36	0.103	-2.62	3-Oxobutanoic acid
**0.038**	-1.43	0.207	-1.52	Gluconic acid, ç-lactone, 5-methoximine
**0.039**	-1.08	0.981	-1.02	Threonine
**0.041**	-1.47	0.619	1.03	Galacturonic acid
**0.042**	2.82	**0.009**	2.66	Propanedioic acid, hydroxyl
**0.047**	-1.14	0.287	-1.06	Proline
**0.048**	-1.16	0.518	1.04	Phenylalanine
**0.048**	-1.04	0.615	1.01	Hexestrol
**0.049**	1.04	0.904	-1.00	2-Butenedioic acid (Z)-
0.459	-1.13	**0.005**	2.48	1-Iodo-2-methylundecane
0.506	1.01	**0.003**	1.21	b-Sitosterol
0.577	1.05	**0.031**	-1.07	1,11-dihydroxyundecane
0.762	-1.02	**0.003**	-1.05	2,6-Bis(1,1-dimethylethyl)-4-methyl-4-methoxycyclohexa-2,5-dien-1-one
0.852	1.01	**0.028**	2.76	Urea
0.862	-1.10	**0.023**	1.47	Indole-3-lactic acid
0.330	1.08	0.762	1.21	TCA
0.488	1.16	0.242	1.10	TDCA
0.967	2.67	0.634	1.00	UDCA
0.157	-1.33	0.269	-1.17	CDCA
0.763	1.29	0.932	1.32	DCA
0.361	2.40	0.533	2.00	GCA
0.700	1.62	0.907	1.44	GUDGA
0.374	-1.14	0.304	1.00	GLCA
0.826	1.29	0.525	2.34	GDCA
0.827	1.11	0.778	1.40	GCDCA

Supplementary Table 6. Metabolite fluxes (or fold changes*) across the splanchnic bed which significantly correlate with the amount of liver fat based on the measurements (without modelling).

**Metabolite**	**Pearson correlation**<br>**(liver fat *vs*. metabolite flux or fold change*)**
Basal state	
Glucose	-0.966
Lactic acid	-0.962
L-valine	-0.954
Leucine	-0.946
2-Ethyl-3-hydroxybutyric acid	-0.946
Pyruvic acid	-0.939
Alanine	-0.876
Succinic acid	-0.872
Cholesterol	-0.841
Azelaic acid	-0.774
Arabifuranose	-0.747
β-Sitosterol	-0.708
Tricarballyic acid	0.712
Methione	0.727
1H-Indelo-3-acetic acid	0.729
3-Hydroxybenzoic acid	0.730
Aminomaloic acid	0.735
l-Tryphtophan	0.765
Urea	0.778
Citric acid	0.788
Phenylalanine	0.791
Glutamine	0.824
Creatinine	0.846
l-Proline, 4-hydroxy	0.848
Altronic acid, lactone	0.849

Butanedioic acid, methylene	0.876
Decanoic acid	0.848
Hexadecanoic acid	0.880
9-Tetradecenoic acid	0.883
2,3,4-Trihydroxybutyric acid	0.892
Ethanolamine	0.921
Glyceryl-3-phosphate	0.924
Butanal, 2,3,4-hydroxy	0.926
Butane, 2,3-hydroxy	0.929
Threonic acid	0.940
Aspartic acid	0.950
Eicosanoic acid	0.954
Oleic acid	0.962
Glyceric acid-3-phosphate	0.971
Docosahexenoic acid	0.990
Monopalmitin	0.995
Low-dose insulin	
α-Hydroxybutyric acid	-0.945
Stearic acid	-0.859
Ornithine	-0.842
4-Methyl-2-oxovaleric acid	-0.818
Adipic acid	-0.789
Cinnamic acid	0.753
Citric acid	0.819

*For metabolites (marked with *cursive*) which were not quantified (*i.e.*, were determined semi-quantitatively), fold changes were used instead of fluxes. For quantified metabolites we found that there is practically no difference in correlation values if fluxes or fold changes are used.

Supplementary Table 7. Reactions whose maximal flux rate in the basal state is correlated with liver fat levels, according to the flux-based modelling.

		Spearman (empirical)		Maximal flux rate at different fat levels				
**Reaction**	**Subsystem**	***P*-value***	**Correlation**	**5**	**30**	**65**	**70**	**70**
pyruvate carboxylase	Pyruvate Metabolism	0.033 (8.26E-03)	0.975	0	86.4	524.98	814.97	1000
dimethylglycine dehydrogenase, mitochondrial	Glycine, Serine, and Threonine Metabolism	0.033 (8.26E-03)	0.975	25	50	63.76	84.28	191.83
Sarcosine dehydrogenase (m)	Urea cycle/amino group metabolism	0.033 (8.26E-03)	0.975	25	50	100.43	168.56	507.72
guanylate kinase (GMP:ATP)	Nucleotides	0.033 (8.26E-03)	0.975	0	57.35	97.62	143.72	1000
5'-nucleotidase (GMP)	Nucleotides	0.033 (8.26E-03)	0.975	0	7.35	97.62	156.69	1000
pyruvate mitochondrial transport via proton symport	Transport, Mitochondrial	0.033 (8.26E-03)	0.975	-201.61	-79.61	517.9	648.04	1000
1-acylglycerol-3-phosphate O-acyltransferase 1	Triacylglycerol Synthesis	0.033 (8.26E-03)	0.975	21.77	83.99	342.06	613.06	443.46
Deoxyadenosine deaminase	Nucleotides	0.033 (8.26E-03)	0.975	101.92	126.79	150	252.64	1000
glucose 6-phosphate endoplasmic reticular transport via diffusion	Transport, Endoplasmic Reticular	0.033 (8.26E-03)	0.975	0	49.45	50.99	210.4	1000
L-proline reversible transport via proton symport	Transport, Extracellular	0.033 (8.26E-03)	1.000	863.6	982.77	998.46	1000	1000

formaldehyde transport via diffusion (mitochondrial)	Transport, Mitochondrial	0.033 (8.26E-03)	0.975	25	50	63.76	84.28	191.83
5'-nucleotidase (dGMP)	Nucleotides	0.033 (8.26E-03)	0.975	50	61.44	150.02	158.99	1000
CO2 transport (diffusion), mitochondrial	Transport, Mitochondrial	0.033 (8.26E-03)	0.975	149.82	249.81	568.21	925.3	1000
Inosine transport (diffusion)	Transport, Extracellular	0.033 (8.26E-03)	0.975	51.92	76.79	100	202.64	1000
purine-nucleoside phosphorylase (Deoxyinosine)	Purine Catabolism	0.033 (8.26E-03)	0.975	51.92	76.79	100	202.64	1000
methenyltetrahydrifikate cyclohydrolase, mitochondrial	Folate Metabolism	0.033 (8.26E-03)	0.975	25	50	100.43	168.56	666.78
Proline transport (sodium symport) (2:1)	Transport, Extracellular	0.033 (8.26E-03)	1.000	863.6	982.77	998.46	1000	1000
betaine-homocysteine S-methyltransferase	Glycine, Serine, and Threonine Metabolism	0.033 (8.26E-03)	0.975	25	50	63.76	84.28	191.83
H2O endoplasmic reticulum transport	Transport, Endoplasmic Reticular	0.033 (8.26E-03)	0.975	535.8	559.34	600.5	846.6	1000
3-oxoacid CoA-transferase	Valine, Leucine, and Isoleucine Metabolism	0.033 (8.26E-03)	0.975	0	98.48	137.83	373.71	1000
Citrate exchange		0.033 (8.26E-03)	1.000	466.51	638.71	832.58	1000	1000
retinol acyltransferase	Vitamin A Metabolism	0.033 (8.26E-03)	0.975	0	50	301.15	1000	551.86
R total flux	R Group Synthesis	0.033 (8.26E-03)	0.975	0	50	50.41	106.25	384.81
dimethylglycine transport via diffusion (cytosol to mitochondria)	Transport, Mitochondrial	0.033 (8.26E-03)	0.975	25	50	63.76	84.28	191.83

phosphoglycerate mutase	Glycolysis/Gluconeogenesis	0.033 (8.26E-03)	0.975	-102.73	0	695.05	995.07	1000
monoacylglycerol acyltransferase	Triacylglycerol Synthesis	0.033 (8.26E-03)	0.975	0	52.71	299.16	1000	555.29
formaldehyde dehydrogenase	Tyrosine metabolism	0.033 (8.26E-03)	0.975	-2.32	100	113.76	134.28	288.96
glutamine-fructose-6-phosphate transaminase	Aminosugar Metabolism	0.033 (8.26E-03)	0.975	0	38.23	210.9	222.97	1000
glycerol kinase	Glycerophospholipid Metabolism	0.033 (8.26E-03)	0.975	0	29.87	198.25	607.39	295.91
deoxyinosine transport via diffusion	Transport, Extracellular	0.033 (8.26E-03)	0.975	51.92	76.79	100	202.64	1000
Deoxyinosine exchange		0.033 (8.26E-03)	0.975	338.05	527.74	728.4	891.31	1000
Deoxyadenosine deaminase, extracellular	Nucleotides	0.033 (8.26E-03)	0.975	101.92	126.79	150	252.64	1000
L-carnitine transport out of mitochondria via diffusion	Transport, Mitochondrial	0.033 (8.26E-03)	0.975	0	2.95	7.9	262.41	1000
formate-tetrahydrofolate ligase	Folate Metabolism	0.033 (8.26E-03)	1.000	-52.32	137.11	776.91	1000	1000
Adenosine deaminase, extracellular	Nucleotides	0.033 (8.26E-03)	0.975	101.92	126.79	150	252.64	1000
S-Formylglutathione hydralase	Tyrosine metabolism	0.033 (8.26E-03)	0.975	-2.32	100	113.76	134.28	288.96
Adenosine deaminase	Purine Catabolism	0.033 (8.26E-03)	0.975	101.92	126.79	150	252.64	1000
glycerol-3-phosphate acyltransferase	Triacylglycerol Synthesis	0.033 (8.26E-03)	0.975	21.77	83.99	342.06	613.06	443.46
glucose-6-phosphate phosphatase, edoplasmic	Glycolysis/Gluconeogenesis	0.033 (8.26E-03)	0.975	0	49.45	50.99	210.4	1000

reticular								

*Corrected for multiple hypothesis *via* FDR, with false discovery rate limited to 0.1.
